# Modulation of osteogenic and myogenic differentiation by a phytoestrogen formononetin via p38MAPK-dependent JAK-STAT and Smad-1/5/8 signaling pathways in mouse myogenic progenitor cells

**DOI:** 10.1038/s41598-019-45793-w

**Published:** 2019-06-26

**Authors:** Ilavenil Soundharrajan, Da Hye Kim, Palaniselvam Kuppusamy, Ki Choon Choi

**Affiliations:** 1 Grassland and Forage Division, National Institute of Animal Science, Rural Development Administration, Cheonan, 31000 Republic of Korea; 2 Center for Research on Environmental Disease, College of Medicine, University of Kentucky, 1095 VA Drive, Lexington, KY 40536 USA

**Keywords:** Drug discovery, Drug development

## Abstract

Formononetin (FN), a typical phytoestrogen has attracted substantial attention as a novel agent because of its diverse biological activities including, osteogenic differentiation. However, the molecular mechanisms underlying osteogenic and myogenic differentiation by FN in C2C12 progenitor cells remain unknown. Therefore the objective of the current study was to investigate the action of FN on myogenic and osteogenic differentiation and its impact on signaling pathways in C2C12 cells. FN significantly increased myogenic markers such as Myogenin, myosin heavy chains, and myogenic differentiation 1 (MyoD). In addition, the expression of osteogenic specific genes alkaline phosphatase (ALP), Run-related transcription factor 2(RUNX2), and osteocalcin (OCN) were up-regulated by FN treatment. Moreover, FN enhanced the ALP level, calcium deposition and the expression of bone morphogenetic protein isoform (BMPs). Signal transduction pathways mediated by p38 mitogen-activated protein kinase (p38MAPK), extracellular signal-related kinases (ERKs), protein kinase B (Akt), Janus kinases (JAKs), and signal transducer activator of transcription proteins (STATs) in myogenic and osteogenic differentiation after FN treatment were also examined. FN treatment activates myogenic differentiation by increasing p38MAPK and decreasing JAK1-STAT1 phosphorylation levels, while osteogenic induction was enhanced by p38MAPK dependent Smad, 1/5/8 signaling pathways in C2C12 progenitor cells.

## Introduction

Development of skeletal muscle cells is a strictly regulated process with diverse functions in organisms. Myogenesis process can be divided into many different phases^1^. Mesoderm-derived structures generate the first muscle fibers of the body. Proper and subsequent waves of additional fibers are generated along these template fibers during embryonic myogenesis^2^. Skeletal muscle cells constitute 40% of the human body and play multiple roles in locomotion and whole body metabolism. Muscle cells play a major role in energy production. Muscle cells utilize higher than 70% of glucose and maintain lipid homeostasis. In addition, they maintain bone homeostasis via bone remodeling. They coordinate osteoblast-mediated bone formation with osteoclast-mediated bone resorption^3^. In general, myogenic regulatory factors (MRFs) play an essential role in the fusion of muscle^4,5^. Especially, basic helix-loop-helix (bHLH) transcription factor, myogenin, myogenic differentiation-1 (MyoD), myogenic factors-5 (Myf5) and myogenic regulatory factor -4 are mainly involved in muscle development. In addition, different intracellular signaling pathways such as p38 MAPK^6,7^, ERK/MAPK^8^, PI3K/AKT^9,10^, BMPs^11^ and JAKs-STATs^12^ regulate osteogenic and myogenic differentiation mediated by specific proteins via hormones, cytokines and growth factor productions^8,13–18^.

Current treatment for osteoporosis is based on the use of anti-resorptive and bone-forming drugs. At the same time, the continuous use of these drugs is highly associated with severe side effects. Therefore, effective treatment approaches without side effects are urgently required for the enhancement of osteoblast and myogenic differentiation. Worldwide several researchers have attempted to identify lead compounds based on natural products that activate osteoblasts^19–23^. Formononetin (FN) is a naturally occurring isoflavone occurring in many natural sources including *Astragalus membranaceus*, *Trifolium pretense*, *Glycyrrhiza glabra, Pueraria lobate* and *Italian ryegrass*. It considered a typical phytoestrogen which found predominantly in the red clover plant^24^. FN shows diverse biological functions^25^. It acts as a neuroprotective^26^, and cardioprotective agent^27^. Furthermore, FN has anticancer effects in lung^28^, colorectal^29^, and prostate cancers^30,31^. Also, FN reduces insulin resistance and hyperglycemia^32^. FN influences the growth and immunological activities in broilers^33^. FN treatment promotes early fracture healing and osteogenic potential through increasing vascular endothelial growth factor (VEGF), VEGF receptor-2 and osteogenic specific markers in a rat fracture animal model^34^. Another report claimed that the high-fat-diet-induced (HFD) obese mice treated with FN at different concentrations exhibited enhanced osteoblast differentiation via restoring the mineralizing capacities and increasing myoblast specific markers such as collagen type-1, RUNX2 and OCN in BMSCs cells than the BMSCs cells obtained from HFD alone treated mice. Also, FN treatment decreased adipogenic potential in obese mice^35^. However, the molecular mechanisms underlying the osteogenic and myogenic enhancement of FN in C2C12 mouse progenitor cells remain unclear. Hence, this study aimed to identify the molecular action of FN in osteogenic and myogenic differentiation and its impact on signal transduction pathways in mouse C2C12 progenitor cells.

## Results

### Effect of Formononetin (FN) on the viability of C2C12 progenitor cells

**Figure 1:**
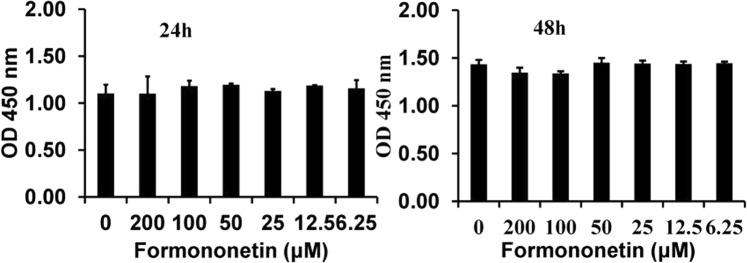
Effect of FN on cell viabilities measured with an Ez-cytox reagent. Cell viability was assayed with an Ez-cytox reagent. At concentration below 50 µM, FN did not significantly affect the cell viabilities of C2C12 after 24 h of treatment. However, at concentrations greater than 50 µM, FN slightly affected their viability after 48 h of treatment without significant (p < 0.05). The results are expressed as the mean ± SEM of six replicates.

Different concentrations of FN on the viability of C2C12 progenitor cells at 24 and 48 h are presented in Fig. 1. At the concentration below 50 µM of FN did not affect cell viabilities at 24 h and 48 h, compared to control cells, whereas, at the concentration greater than 50 µM, FN slightly affected cell viability after 48 h of treatment. However, there is no statistical significance (p < 0.05) between all concentrations of FN and control cells. At the same time, FN at the concentration of less than 50 µM is the safer concentration for further experiments.

### FN promotes both osteogenic and myogenic potential in C2C12 progenitor cells

**Figure 2:**
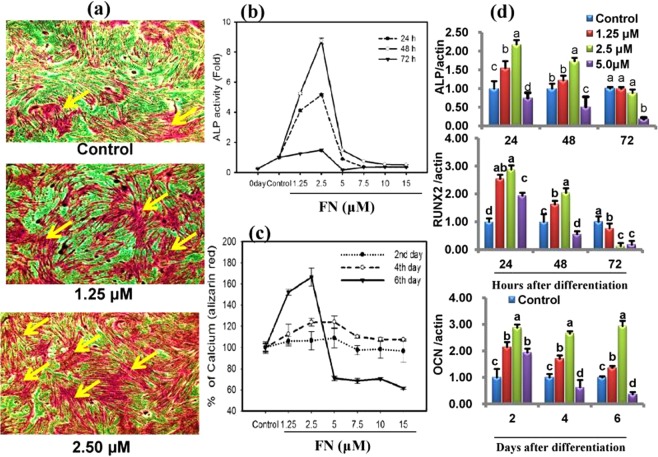
Increased ALP activity, calcium deposition and its markers gene by FN. Osteoblast differentiation was induced with osteogenic induction medium (HSCG) in the presence of different concentrations (1–15 µM) of FN and incubated for different days. (**a**) Cells were fixed in formalin and stained with Alizarin Red S for 30 min. Cell images were obtained by EVOS cell image system at 10X. (**b**) Cell extracts were prepared and used for ALP activity at 24, 48, and 72 h. (**c**) Alizarin Red S stain was extracted and quantified on day 2, 4 and 6 according to the kit protocol. (**d**) The total RNA was extracted and reverse transcribed for quantification of mRNA by qPCR. ALP, RUNX2 and osteocalcin mRNA levels were determined after normalization with β-actin. All values represent mean ± SEM, n = 6 for ALP, calcium level and qPCR. Different letters a, b, c and d within a column indicate significant differences between treatment and non-treatment (p < 0.05). Statistical significance was performed using the general linear model with multivariate, post hoc test and comparisons with respective controls.

**Figure 3:**
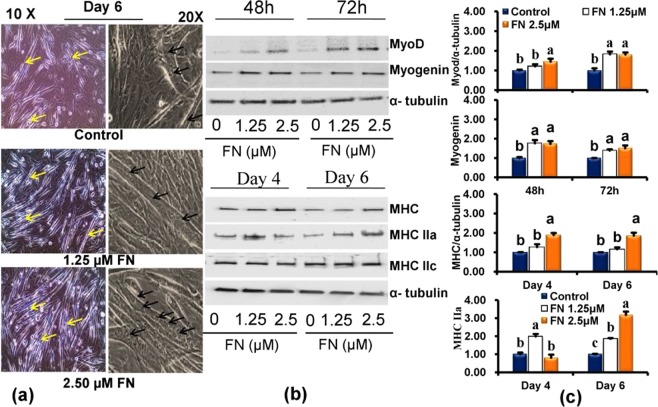
FN treatment potently increased multinucleated myotube formation and its specific makers in the HSCG medium. (**a**) Multinucleated myotubes were captured by EVOS cell image system at 10X and 20X. (**b**) Protein lysates were prepared and used for immunoblotting using specific antibodies against key myogenic markers myoD, myogenin, myosin heavy chains (MHC) and α-tubulin. (**c**) The intensity of protein reacted bands was determined by densitometry using ImageJ software. Bars represent mean ± SEM for three replicates and statistical analysis was performed using a general linear model with multivariate, post hoc test and comparisons with respective controls. *p* < 0.05 level was considered significance between treatment and non-treatment.

First, we analyzed whether FN treatment promoted osteogenic and myogenic activities in the presence of 2% horse serum with 50 µg/mL Vitamin C and 10 mM β-glycerophosphate (HSCG). FN treatment potentially increased the levels of early and delayed osteogenic markers such as ALP at 24, 48, 72 h, and calcium deposition at days 2, 4, and 6 (Fig. 2a–c). At the same time, microscopic views showed higher numbers of multinucleated myotubes than the control cells (Fig. 3a). It prompted us to investigate the osteogenic and myogenic properties of FN simultaneously in C2C12 cells cultured in the same medium.

### FN treatment enhanced the expression of osteogenic and myogenic markers

FN treatment potently enhanced both osteogenic and myogenic differentiation. Therefore, we investigated the expression of osteogenic specific transcripts, ALP, osteocalcin, RUNX_2_ and the expression of specific myogenic markers myoD, myogenin, myosin heavy chain and its isoforms (MHC) at different time intervals. Quantitative-PCR analysis revealed that HSCG significantly upregulated the osteoblast markers during differentiation, while FN combined with HSCG potently enhanced the transcription of ALP, OCN, and RUNX_2_ (Fig. 2d). The ALP and RUNX_2_ expression levels were peaked at 24 h, whereas the OCN expression was upregulated throughout experimental periods after FN treatment at a concentration of 2.5 µM (p < 0.05).Western blot results indicated that FN treatment strongly increased the expression of myogenic markers including myoD, myogenin, and myosin heavy chain and its isoforms (MHC) at different time intervals during differentiation (Fig. 3b,c).

### FN treatment Increased bone morphogenetic protein isoforms (BMPs)

**Figure 4:**
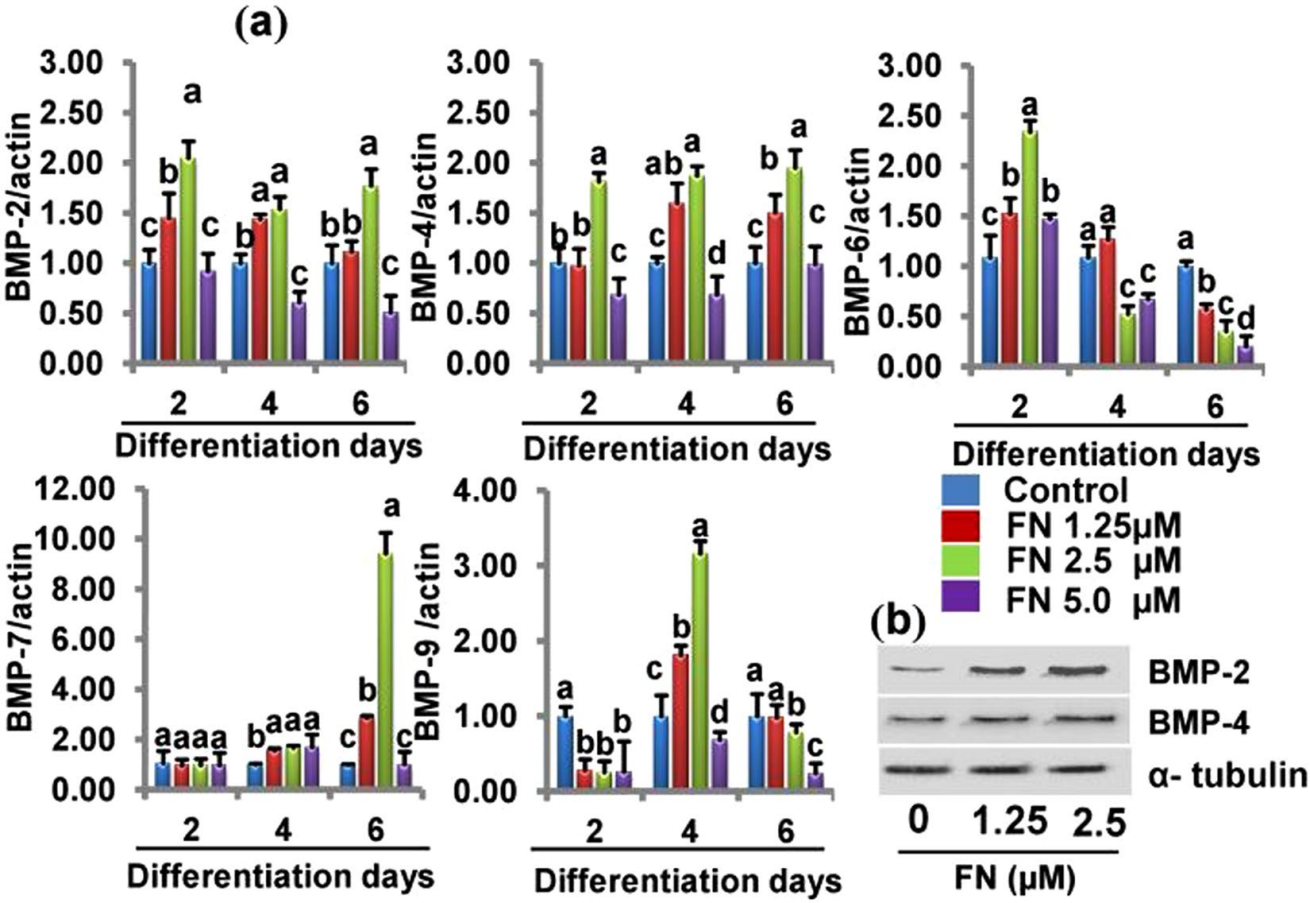
Effects of FN on the expression of bone morphogenetic proteins (BMPs) in experimental cells. Cells were differentiated with HSCG medium in the presence/absence of different concentration of FN (1.25–5 µM) for 6 days. (**a**) BMPs mRNA expression in the experimental cells at different time points. (**b**) Levels of BMP-2 and BMP-4 protein expression in the experimental cells on day 6. Bars display mean ± SEM of six experimental replicates. Different letters a, b, c, and d within a column indicates significant differences between groups (p < 0.05). Statistical significance was performed using a general linear model with multivariate, post hoc test and comparisons with respective controls.

Next, we investigated the osteogenic potential and enhancement of different forms of BMPs by FN *in vitro*. We found that FN treatment potently upregulated the genes encoding BMP-2, BMP- 4, BMP- 6 BMP-7, and BMP-9 compared with the control cells. The induction of BMP-2, BMP- 4 and BMP-7 was accelerated by FN until day 6, whereas the levels of BMP- 6 and BMP-9 were upregulated by day 2 and 4, respectively (Fig. 4a). Western blot results revealed that BMP-2 and BMP-4 protein expression was also induced after FN treatment compared with control on day 6 (Fig. 4b). The results indicate that FN treatment induced osteogenic differentiation in C2C12 cells via induction of different BMPs at different time points.

### FN regulates different signaling pathways mediating osteogenic and myogenic differentiation

**Figure 5:**
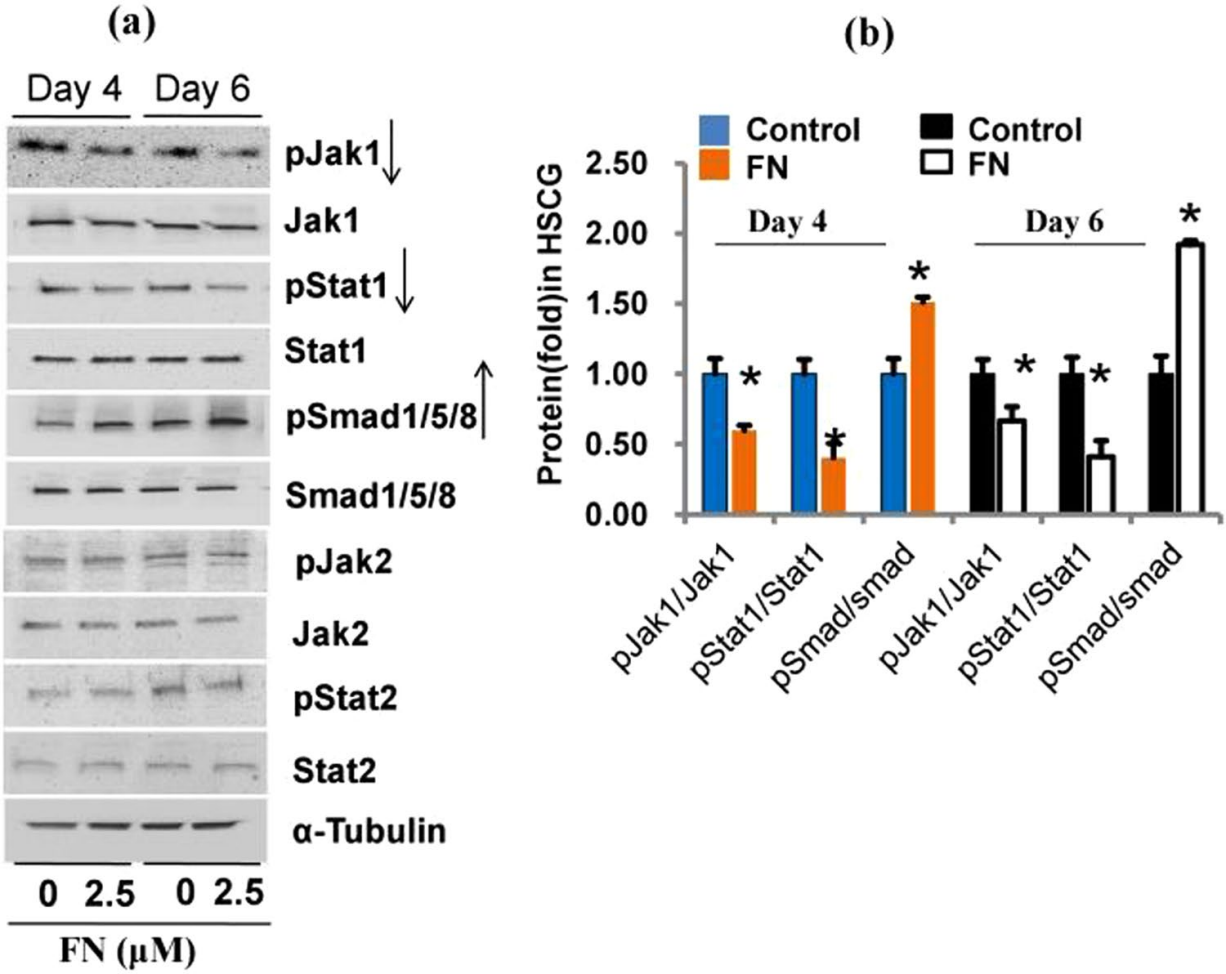
FN role in JAKs-STATs and Smad1/5/8 signaling pathways in experimental cells. Cells were treated with FN in HSCG media for six days. Proteins were harvested and the phosphorylation levels of JAKs-STATs and Smad 1/5/8 proteins were analyzed on day 4 and 6 by immunoblotting using specific antibodies against targets. (**a**) JAKs-STATs and Smad 1/5/8 phosphorylation level after treatment with FN in HSCG medium. (**b**)The intensity of protein bands was quantified by densitometry using ImageJ software. Bars display mean ± SEM of three experimental replicates. *p < 0.05 represents a statistically significant difference between control and treatment.

As FN showed both osteogenic and myogenic potential in the HSCG medium, we investigated the mechanism of FN-mediated regulation of signal transduction pathways involved in osteogenic and myogenic differentiation of C2C12 progenitor cells. First, we analyzed the phosphorylation levels of JAK1, JAK2, STAT1, STAT2 and Smad 1/5/8 in the differentiated cells on day 4 and 6 after FN treatment in the same medium (Fig. 5a,b). FN treatment decreased JAK1/STAT1 and increased phosphorylated Smad 1/5/8 levels compared with the control cells. However, JAK2/STAT2 phosphorylation was similar in control and FN-treated cells. We then decided to investigate the precise mechanisms underlying osteogenic and myogenic differentiation. For myogenic signaling confirmation, cells were treated with FN at a concentration of 2.5 µM in the myogenic differentiation medium containing 2% horse serum (HS) for 6 days. Results suggested that FN treatment downregulated the phosphorylated levels of JAK1/STAT1 compared with the control, while the Smad 1/5/8 phosphorylation was not altered significantly between control and FN treatment in HS medium (Supplementary Fig. S1). It confirmed that FN treatment increased osteogenic differentiation by increasing the levels of Smad 1/5/8 phosphorylation while myogenic differentiation was promoted by decreasing the JAK1/STAT1 phosphorylation compared with control cells.

**Figure 6:**
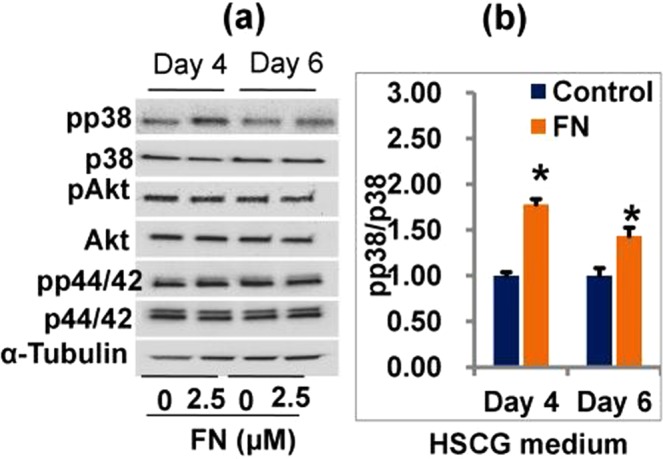
Effect of FN on p38MAPK, AKT and p44/42 signaling pathways in experimental cells. Cells were treated with 2.5 μM FN in the presence of HSCG media for six days. Proteins were then extracted and analyzed by immunoblotting using specific antibodies against p38MAPK, AKT, and p44/42. (**a**) Regulation of p38MAPK, AKT, and p44/42 signaling by FN treatment. (**b**) The intensity of protein bands was determined by ImageJ software. Bars display mean ± SEM of three experimental replicates. *p < 0.05 indicates a statistically significant difference between treatment and non-treatment.

**Figure 7:**
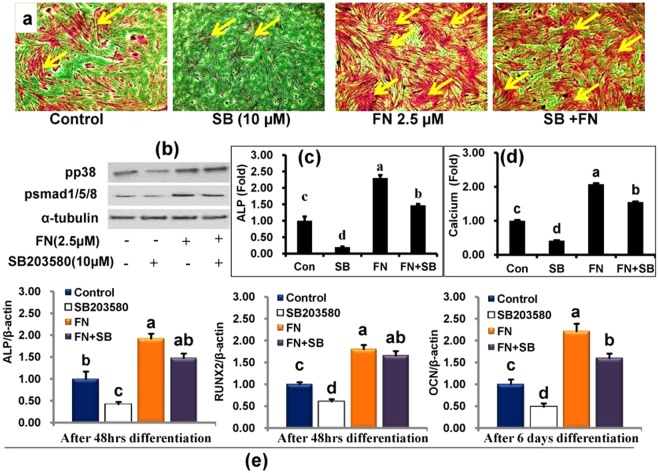
A competitive study between FN (2.5 μM) and p38 inhibitor (10 μM) upon osteogenic enhancement of C2C12 cells. Cells treated with FN in the presence/absence of SB203580 (10 µM) for 48 h. (**a**) Experimental cells were fixed and stained with Alizarin Red S for 30 min. Cell images were obtained using the EVOS cell image system at 10X, arrows indicate calcium deposition in cells. (**b**) Proteins were extracted and separated by SDS-PAGE for immunoblotting with antibodies against pp38, pSmad1/5/8 and α-tubulin. (**c**) Cell extracts were prepared and used for the determination of ALP activity at 48 h. (**d**) Cells stained with Alizarin Red S stain were extracted and quantified on day 6 according to the kit protocol. (**e**) RNA was extracted from the experimental cells and subjected to cDNA synthesis for quantification of ALP, RUNX2 and osteocalcin mRNA expression. All values represent mean ± SEM, n = 6 for ALP, calcium level and qPCR. Different letters a, b, c and d within a column indicate significant differences between treatment and non-treatment groups (p < 0.05).

**Figure 8:**
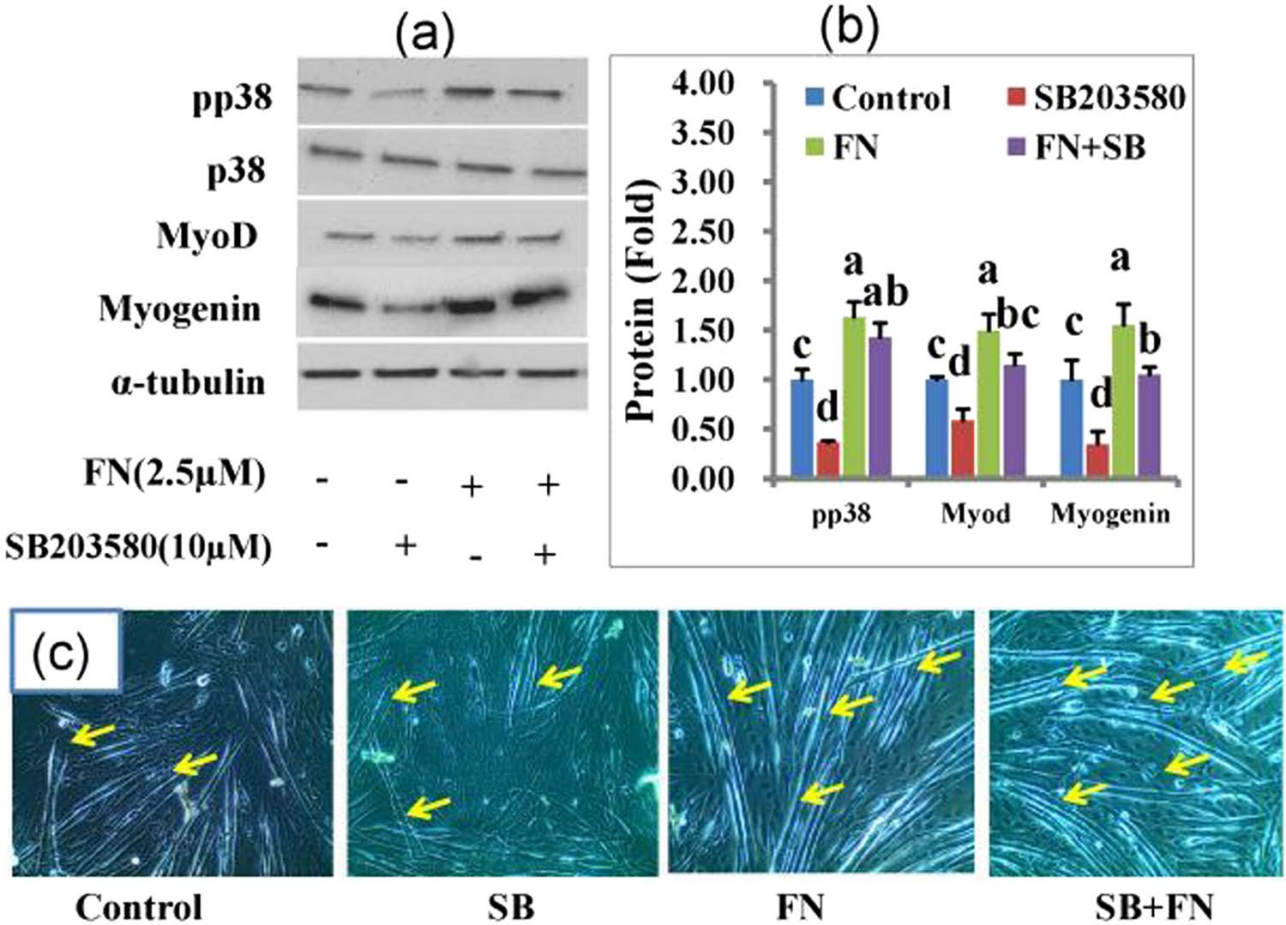
A competitive study between FN (2.5 μM) and p38 inhibitor (10 μM) upon myogenic enhancement in C2C12 cells. Cells treated with FN in the presence/absence of SB203580 (10 µM) for 48 h. Proteins were resolved by SDS-PAGE and incubated with specific antibodies targeted against pp38, myoD, myogenin, and α-tubulin for immunoblotting analysis. (**a**) Key myogenic proteins expression in the experimental cells. (**b**) Quantitative analysis of protein bands was performed using the ImageJ program. (**c**) Myotubes formation in the experimental cells. Bars represent mean ± SEM of three experimental replicates. Different letters a, b, c and d within a column indicate significant differences between treatment and non-treatment groups (p < 0.05).

Finally, we explored the role of p38MAPK, AKT, and ERK 1/2 pathways in FN-induced osteoblast and myoblast differentiation. The levels of p38MAPK, AKT, and ERKs phosphorylation were determined by western blot with specific antibodies on day 4 and 6 following FN treatment in HSCG and HS medium (Figs 6a,b and S2). FN treatment at 2.5 µM activated p38MAPK by increasing its phosphorylation level, while the levels of p44/42 and AKT were not altered by FN when compared with the control cells. Therefore, a specific p38MAPK inhibitor was used in this study to establish FN-induced osteogenic and myogenic differentiation. Cells treated with p38 inhibitor alone at 10 μM in HSCG medium for 48 h decreased ALP activity, calcium deposition, and osteogenic specific genes such as ALP, RUNX_2_ and osteocalcin mRNA expression compared with the control and FN-treated cells. FN treatment combined with p38 inhibitor increased ALP activity, calcium deposition and osteogenic specific genes ALP, RUNX_2_ and osteocalcin mRNA expression compared with the control and cells treated with p38 inhibitor alone (Fig. 7a–e.). In addition, cells treated with p38 inhibitor showed the reduced development of multinucleated myotubes and myogenic specific markers myoD, and myogenin proteins whereas treatment of p38 inhibitor together with FN at 2.5 μM reversed the p38 inhibitor-mediated inhibition of myotube formation and its associated myogenic markers compared with control and p38 inhibitor-treated cells in HSCG (Fig. 8a–c). Overall results suggest that FN treatment stimulated osteoblast differentiation by activating p38MAPK/Smad/BMP signaling pathways, while myogenic differentiation was regulated by p38MAPK/JAK1/STAT1 signaling. The key findings suggest that p38MAPK plays a significant role in both osteogenic and myogenic enhancement of C2C12 progenitor cells by FN.

## Discussion

FN is a phytoestrogen known to display a diverse spectrum of biological activities. Its functional role in osteogenic and myogenic differentiation is poorly demonstrated. Hence, we explored the role of FN in C2C12 cell differentiation into osteogenic and myogenic lineage as well as the underlying signal transduction pathways. FN showed a strong osteogenic and myogenic potential in C2C12 progenitor cells. Enhancement of alkaline phosphatase, OCN, RUNX and other factors such as BMPs, is important for the differentiation of C2C12 progenitor cells into pre-osteoblasts. In mouse C2C12 cells, BMP- 2, 4, 6, 7, and 9 strongly induced the expression of early osteogenic marker ALP and late osteogenic marker OCN^36^. OCN and osteopontin (OPN) are osteogenic markers mediating the intermediate to late stages of osteogenesis. They enhance calcium deposition in the late phases of osteogenesis^37^. The current study findings strongly reinforce the results discussed above. Indeed, FN treatment increased ALP production and its mRNA expression in C2C12 cells in a time-dependent manner. ALP and its mRNA expression were increased until 48hrs by FN treatment, whereas later, the levels of ALP and its mRNA expression were downregulated. ALP has been widely accepted to be a strong and early marker of osteoblast differentiation. It is responsible for the mineralization of the extracellular matrix (ECM)^38^. In addition, calcium deposition and the gene encoding OCN at later stages of differentiation were accelerated by FN treatment as compared with the control. Osteopontin and OCN are commonly used as early and late markers of osteogenic differentiation respectively^39^.

FN treatment increased myogenic differentiation via upregulation of specific myogenic markers in a time-dependent manner. Differentiation of myogenic cells is a highly organized process that is regulated by the members of MyoD family including, MyoD and myogenin as well as the proteins in the myocyte enhancer factor (MEF2) family. The process of differentiation is highly complex and involves cell cycle termination, expression of myogenic specific genes, and multinucleate myotube formation^2,40,41^. Myogenin plays an essential role in myoblast differentiation. It acts at later stages of myogenesis to control their fusion^42^. The terminal differentiation of myoblasts into skeletal myocytes and fusion into myotubes is mediated by the controlled increase in the expression of MyoD, Myf5, myogenin and MRF4, and the decreased the activity of cell cycle regulatory factors^43^. The activation of myogenic regulatory factors (MRFs) including MyoD, myogenic factor 5 (Myf5), MRF4, and myogenin, also regulates the expression of several muscle-specific genes, such as myosin heavy chain (MyHC) and creatinine kinase in muscle fiber type maturation^44–46^.

Accordingly, FN treatment increased the formation of multinucleated myotubes in C2C12 cells compared with the control, without affecting the cell viability or morphology. These results based on cell morphology and analysis of specific myogenic markers suggests that FN increased the fusion of myoblasts into multinucleated myotubes. Myogenin is a key factor required for muscle cell differentiation^45^. Its level is increased during the early stages of differentiation, and decreased in fully differentiated cells. Also, MyHC and its isoforms are increased during different stages of cellular differentiation^47^. The expression of myogenic specific proteins such as myoD, myogenin, and muscle-specific genes myosin heavy chain and myosin heavy chain IIA during the differentiation phase was analyzed. Our data suggested that FN treatment increased the expression levels of myoD, myogenin, myosin heavy chain and myosin heavy chain IIA in differentiated cells compared with the control cells.

Bone morphogenetic protein induction and Smad1/5/8 signaling in osteogenic differentiation by FN were examined, because BMPs are known to mediate bone formation via Smad signaling^21,48,49^. BMPs including, BMP-2, BMP-4, BMP-6, BMP-7, and BMP-9 promote osteoblast differentiation of mesenchymal stem cells. BMP-2^50^, BMP-7, BMP-4, BMP-6, and BMP-9 exhibit strong osteogenic potential in C2C12 cells^51^. BMP-2 and BMP-7 induce rapid bone formation and increase the endogenous expression of BMP-4^52^. BMP9 is recognized as one of the most osteogenic BMPs. It promotes osteoblastic differentiation of mesenchymal stem cells (MSCs) both *in vitro* and *in vivo*^36,53–57^. Bone morphogenetic protein -9 might provides a useful clinical strategy for the augmentation of bone regeneration and healing compared with other BMPs^11^. FN treatment significantly accelerated the expression of BMP-2, BMP-4, BMP-6, BMP-7, and BMP-9. Furthermore, the levels of Smad 1/5/8 phosphorylation were increased compared with the control cells. Osteogenic activities of BMPs are known to activate Smad-Runx2^58^. Our results corroborate the findings of previous reports suggesting that FN treatment activates the Smad1/Smad 5/Smad8 expression by increasing its levels of phosphorylation. In addition, FN treatment increased the mRNA expression of Runx2 compared with the control suggesting that FN activated Smads/1/5/8 signaling during osteogenic differentiation by inducing BMP transcriptional activity.

As FN showed both osteogenic and myogenic potential, we investigated its role in the regulation of signaling pathways involved in osteogenic and myogenic differentiation of C2C12 cells. First, we analyzed the phosphorylation levels of JAK1, JAK2, STAT1, and STAT2 in differentiated cells on day 4 and 6 post-treatment with FN. It is known that the JAKs/STATs pathway plays an essential role in myogenic differentiation. The JAK1/STAT1/STAT3 axis is involved in myoblast proliferation, which prevents premature differentiation into myotubes^59^. JAK2/STAT2/STAT3 expression appears to positively regulate differentiation, indicating that STAT3 elicits specific responses at various times during myogenesis. Inhibition of JAK2 expression abrogates myogenic differentiation. At the same time, JAK1 knockdown accelerates myogenic differentiation, while proliferation is inhibited in C2C12 cells and primary myoblasts^60^. In addition, STAT1 knockdown promotes myogenic differentiation in both primary and immortalized myoblasts^59^. Therefore, we analyzed whether FN altered JAK/STAT signaling pathways involved in the differentiation. Our data showed that FN treatment downregulated JAK1/STAT1 expression by decreasing their phosphorylation level. However, the phosphorylation level of JAK2/STAT2 was not altered significantly between control and FN-treated cells. These results confirm that FN regulated myogenic differentiation via inhibition of JAK1/STAT1 by decreasing their phosphorylation.

At last, we determined the role of FN on p38MAPK, AKT and ERK1/2 signaling pathways involved in osteogenic and myogenic differentiation of C2C12 cells. Extracellular signals regulating both osteogenic and myogenic signals are transduced to the nucleus by mitogen- activated-kinases. Inhibition of p38 prevents the differentiation mechanism in myogenic cell lines and human primary myocytes. Inhibition of p38 also prevents induction of early markers such as myogenin, p21, and late (MHC) myogenic markers^8^. In addition, p38 MAPK phosphorylation plays a key role in the regulation of ALP production during MC3T3-E1 cells differentiation. Inhibition of p38 MAPK by specific inhibitors decreased the ALP expression and mineral deposition in MC3T3-E1 cells^61^. Another study reported that p38 MAPK was required for the expression of ALP and osteocalcin while ERKs were necessary for OC expression only^62^. Several investigators have reported that the ERK groups of MAPKs also play a role in myogenic differentiation. While some investigators have indicated that ERK members inhibit differentiation^63,64^ others reported that ERKs are positive regulators of myogenesis^65^. Akt signaling also plays a major function in hypertrophy and contributes to the myotubes size increases in C2C12 cells^21^. In addition, IGF-phosphoinositide 3-kinase (PI3K)-Akt signaling has been shown to induce myogenic differentiation by stimulating genes specific for myogenic markers such as myogenin, MyoD and MEF2^66,67^. The current study demonstrates that treatment of FN significantly increased the p38 MAPK expression at 2.5 μM of FN without altering Akt or ERKs phosphorylation levels. These results suggest that FN enhanced both osteogenic and myogenic induction via p38 signaling without altering Akt or ERKs pathways. Furthermore, the augmentation of the p38 pathway by FN treatment in differentiated cells was investigated using specific inhibitors. Cells were treated with SB-203580; a p38 inhibitor potently reduced both osteogenic and myogenic differentiation by downregulating specific myogenic markers such as myogenin, myoD, and osteogenic markers including the level of ALP, calcium accumulation and the specific gene expression ALP, RUNX2 and OCN in cells. By contrast, FN treatment with p38 inhibitor accelerated both osteogenic and myogenic specific genes and protein expression.

## Conclusion

In conclusion, the current data suggest that FN treatment significantly increases ALP activity, calcium deposition, and the expression of osteogenic key markers including ALP, RUNX2, OCN and myogenic specific genes such as myogenin, MyoD, myosin heavy chains. BMP- 2, BMP-4, BMP-6, BMP-7, and BMP-9 levels were enhanced by the FN treatment in a concentration and time-dependent manner. FN treatment activates myogenic differentiation by increasing p38MAPK and decreasing JAK1-STAT1 phosphorylation levels, while osteogenic differentiation was enhanced by p38MAPK dependent Smad, 1/5/8 signaling pathways in C2C12 progenitor cells. FN might represent a potential lead compound to promote osteogenic and myoblast differentiation in C2C12 progenitor cells, especially regulating the progression of osteogenic enhancement and myotube morphology.

## Methods

### Cell culture and reagents

The C2C12 mouse myogenic progenitor cells line was procured from the American Type Culture Collection [ATCC, Rockwille, MD, USA]. Dulbecco’s modified Eagle’s medium [DMEM-30-2002] and fetal bovine serum (FBS-30-2020) were procured from ATCC [Rockwille, MD, USA]. Kits for mRNA extraction, iScript cDNA synthesis and qPCR were purchased from Bio-Rad [Biorad- California, USA]. FN, Vitamin C and β-glycerophosphate were obtained from Sigma Aldrich (St. Louis, MO, USA). SB203580 inhibitor was provided by Cell Signaling Technology (Danvers, MA, USA). BMP-4 BMP-2, MyoD, Myogenin, α-tubulin, myosin heavy chain (MHC), MHCIIa, MHCIIc, JAK/pJAK1(Tyr1034/1035), JAK2/pJAK2(Tyr1008), STAT1/pSTAT1(Ser727), STAT2/pSTAT2(Tyr690), Smad/Smad1(ser463/465)/5(ser 463/465)/9(Ser465/467), p38MAPK/pp38MAPK (Thr180/Tyr182), ERKs/ERK(Thr202/Tyr204) and Akt/pAkt (Ser473) were acquired from Cell Signaling Technology (Danvers, MA, USA), Abcam (Cambridge,UK) and Santa Cruz Biotechnology (Dallas,Texas, USA).

### Formononetin preparation

FN stock solution was prepared in DMSO. The fresh working FN was prepared in DMEM-30-2002 from the stock FN for every treatment.

### Determination of cell viability

Ez-cytox assay kit (iTSBiO, Seoul, Korea) was used to determine the effects of FN on cell viability. In details, the cells (C2C12-ATCC, USA) at the density of 1 × 10^4^ were treated with different concentrations of FN after 24 h seeding in 96 well plates and incubated at 37 °C with 5% CO_2_ for 24 h and 48 h. After incubation, ten microliters of WST reagent was added to each well and incubated at 37 °C with 5% CO_2_ for 1 to 2 h. The cell viability was measured at 450 nm using a Packard SpectraCount Absorbance Microplate Reader [Packard Instrument Co., Downers Grove, IL].

### Osteogenic differentiation

C2C12 progenitor cells were seeded into 6-well (or) 12-well cell culture plates at a density of 5 × 10^4^ or 2.5 × 10^4^ cells/well, respectively. Cells were cultured in 10% FBS in DMEM (ATCC30-2002) medium and incubated at 37 °C with 5% CO_2_. Osteogenic induction was performed according to the previous method^68^ with modifications. The growth medium was replaced by osteogenic differentiation medium containing vitamin C (50 µg/mL) and β-glycerophosphate (10 mM) in the presence of 2% horse serum (HSCG) medium after cells reached 80–90% confluence. FN at different concentrations was exposed to the cells in the HSCG medium for every 48 h during the experimental periods.

### Myogenic differentiation

C2C12 cells were seeded into 6-well (or) 12-well cell culture plates at a density of 5 × 10^4^ or 2.5 × 10^4^ cells/well, respectively. The cells were cultured in 10% FBS in DMEM DMEM (ATCC30-2002) medium and incubated at 37 °C with 5% CO_2_. Myogenic differentiation was induced with myogenic differentiation medium consisting of DMEM with 2% horse serum (HS) after cells reached 80–90% confluence. The growth medium was replaced by 2% HS medium for every 48 h with different concentration of FN until the end of the experimental periods^69^.

### ALP quantification

Experimental cells were harvested at different time points and washed with cold PBS three times. Cells were suspended in 500 µL of assay buffer and homogenized using a homogenizer. Cell lysates were centrifuged at 12000 g, 4 °C for 15 min. The supernatants were collected and stored on ice for further assay. The alkaline phosphatase activity of samples was measured using the ALP assay kit (Abcam, Cambridge, MA, USA).

### Calcium staining and quantification

The culture medium was aspirated and washed with PBS twice. Cells were fixed with 1 mL of 4% paraformaldehyde in PBS for 15 min at room temperature. Subsequently, after carefully removing the fixative, the cells were washed three times with dH2O. After draining the water completely, 1 mL of 2% Alizarine Red S stain solution was slowly added to each well. Plates were then incubated at the room for 30 min temperature. After removing excess dye, plates were washed 3–5 times with dH_2_O followed by the addition of water 1 mL to each well. Images were obtained using an inverted microscope (CKX41, Olympus Corporation, Tokyo Japan]. Calcium deposition in differentiated cells was quantified using the Alizarin Red S staining quantification kit according to the manufacturer’s protocol (Science Cell, Carlsbad, CA).

### Real-time quantitative reverse transcription PCR

Total RNA of the experimental cells were extracted and quantified using RNeasy lipid mini Kit (Qiagen, MD, USA) and Spectramaxi3(Molecular Devices, California, USA), respectively. Five hundred nanograms of total RNA used to cDNA synthesis using an iScript cDNA synthesis kit (Biorad, California, USA). Gene expression patterns were quantified by SYBR Green-based qPCR using gene-specific primers: ALP(R-gctccacaaacgagaaaagc; F-tccttcacgccacacaagta), BMP-2(R-acgtcctcagcgagtttgag; F-ctctccagccggtggtct), BMP-4 (R-cagcatcccagaaaatgagg; F-ttatacggtggaagccctgt), BMP-6 **(**R**-**gcagcagcagcagcagac; F-ctcttcgtcgtcattggaca),BMP-7(R-gggcttctcctacccctaca; F-tccactaggttgacgaagctc), BMP-9(R-ggagaggagggtgtctttga; F-gttttgtcctgggagggaat), OC(R-agtccccagcccagatcc; F-ccgtagatgcgtttgtaggc); RUNX2 (R-caacagagggcacaagttct; F-gctcggatcccaaaagaag) β-actin (R-tatggaatcctgtggcatcc; F-tggtaccaccagacagcact) on a CFX 96 Real-Time PCR detection system (Biorad, California, USA). Expressions of target genes were quantified after normalization with β-actin^70^.

### Protein extraction and immunoblotting

Proteins lysates were prepared from the cells using Radioimmunoprecipitation assay buffer (RIPA) (Rockland, Limerick, PA) with 1X protease and phosphatase inhibitors (Roche, Basel, Switzerland and Sigma Aldrich, St. Louis, USA). Cells were washed three times with PBS. After the addition of the required volume of RIPA lysis buffer based on the plate types, cells were incubated at 4 °C for 5 min. Cells were scraped rapidly with a cell scraper (TPP, Trasadingen, Switzerland) to remove and lyse residual cells. The cell lysate was transferred to a 2 mL tube and centrifuged at 8000 g for 10 min at 4 °C. Protein concentration was quantified by the Pierce BCA protein assay kit (Thermofisher Scientific, Massachusetts, USA). Protein samples were separated by pre-casting-SDS-PAGE (4–12%, Biorad- California, USA) and blotted onto polyvinylidene difluoride (PVDF) membranes (Trans-blot Turbo transfer system, Biorad, California, USA). Immunoblotting was performed according to the western breeze chemiluminescence kit (Invitrogen, Massachusetts, USA) using rabbit monoclonal and polyclonal antibodies. All primary antibody reactions were carried out at 4 °C for overnight (Cell Signaling Technology antibodies 1:1000; Santa Cruz Biotechnology antibodies 1.500; Abcam antibodies 1:1000) against specific proteins^69^. The HRP-conjugated secondary antibody was used for the detection of primary antibodies (Cell Signaling Technology, Danvers, MA, USA). Band signals were analysed with an enhanced chemiluminescence kit (Bio-Rad- California, USA) on a chemiluminescence imaging system (Davinch gel imaging system, Seoul, South Korea). The intensity of immunoreactive bands was quantified with ImageJ software - 1.49 version(32 bit), (Wayne Rasband, National Institute of Health, USA).

### Statistical analysis

The data generated from experiments were subjected to one-way ANOVA and multivariate comparisons analysis using Statistical Package for the Social Sciences (SPSS-16). Less than 0.05 was considered as Statistical significance between the treatment and non-treatment.

## Supplementary information

Modulation of osteogenic and myogenic differentiation by a phytoestrogen formononetin via p38MAPK-dependent JAK-STAT and Smad-1/5/8 signaling pathways in mouse skeletal muscle cells

## Data Availability

The data generated and analyzed for the current study are available from the corresponding author upon reasonable request.
