# Supplementary material for: Modulation of osteogenic and myogenic differentiation by a phytoestrogen formononetin via p38MAPK-dependent JAK-STAT and Smad-1/5/8 signaling pathways in mouse myogenic progenitor cells
Source: Sci Rep. 2019 Jun 26;9:9307. doi: 10.1038/s41598-019-45793-w (PMC6594940; doi:10.1038/s41598-019-45793-w)
Supplement: Supplementary file 1 — Modulation of osteogenic and myogenic differentiation by a phytoestrogen formononetin via p38MAPK-dependent JAK-STAT and Smad-1/5/8 signaling pathways in mouse skeletal muscle cells [file 41598_2019_45793_MOESM1_ESM.pdf]

Modulation of osteogenic and myogenic differentiation by a phytoestrogen formononetin via p38MAPK-dependent JAK-STAT and Smad-1/5/8 signaling pathways in mouse myogenic progenitor cells

Ilavenil Soundharrajan[1]†, Da Hye Kim[2]†, Palaniselvam Kuppusamy[1], Ki Choon Choi*[1]

[1] Grassland and Forage Division.National Institute of Animal Science. Rural Development Administration. Cheonan. Republic of Korea-31000.

[2]Center for Research on Environmental Disease, College of Medicine, University of Kentucky, 1095 VA Drive, Lexington, KY 40536 USA.

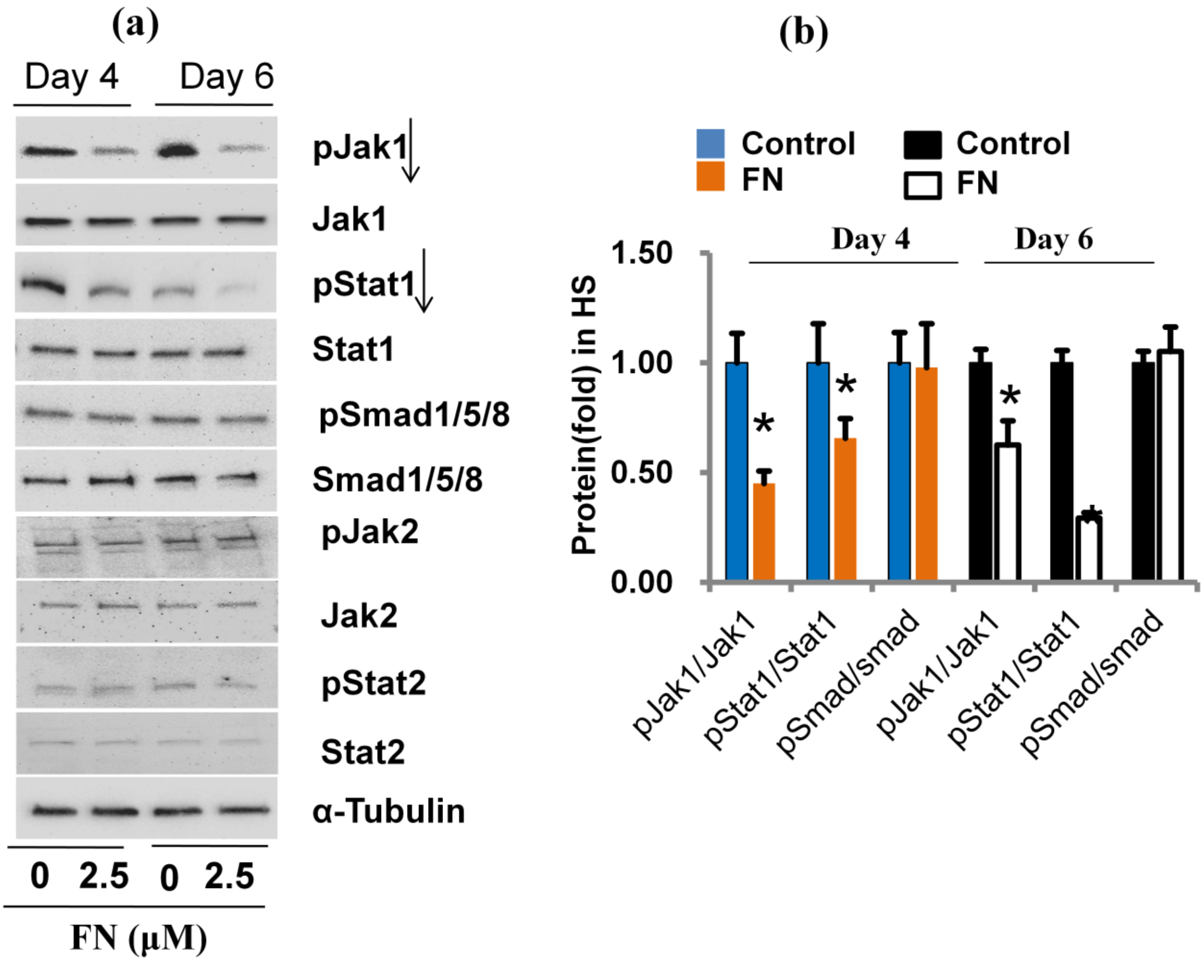

Supplementary Figure S1. FN role in JAKs-STATs and Smad1/5/8 signaling pathways in experimental cells in 2% horse serum medium (HS). Cells were treated with FN in HS medium for six days. Proteins were harvested and the phosphorylation levels of JAKs-STATs and Smad 1/5/8 proteins were analyzed on day 4 and 6 by immunoblotting using specific antibodies against targets. JAK1 and STAT1 phosphorylation level was downregulated while SMAD and JAK2/STAT2 phosphorylation was not altered by FN treatment (a) JAKs-STATs and Smad 1/5/8 phosphorylation level after treatment with FN in HS medium. (b)The intensity of protein bands was quantified by densitometry using Image J software. Bars display mean ± SEM of three experimental replicates. *$p<0.05$ represents a statistically significant difference between control and treatment.

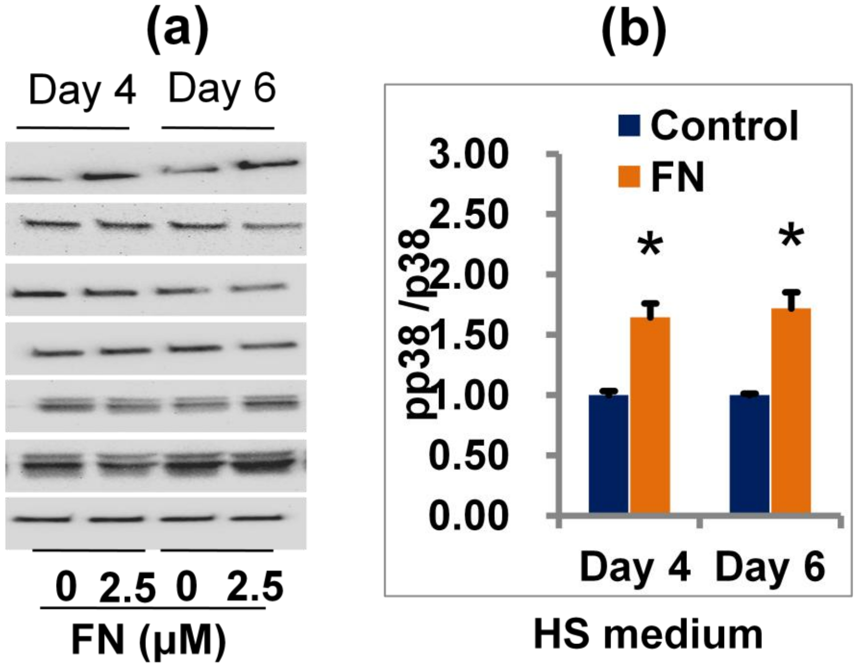

Supplementary Figure S2. Effect of FN on p38MAPK, AKT and p44/42 signaling pathways in experimental cells in HS medium. Cells were treated with 2.5μM FN in the presence of HS medium for six days. Proteins were then extracted and analyzed by immunoblotting using specific antibodies against p38MAPK, AKT, and p44/42 (a) Regulation of p38MAPK, AKT, and p44/42 signaling by FN in HS medium. (b)The intensity of protein bands was determined by Image J software. Bars display mean ± SEM of three experimental replicates. *$p<0.05$ indicates a statistically significant difference between treatment and non-treatment.
